# Human resources for health challenges of public health system reform in Georgia

**DOI:** 10.1186/1478-4491-6-8

**Published:** 2008-05-27

**Authors:** Mamuka Djibuti, George Gotsadze, George Mataradze, George Menabde

**Affiliations:** 1 Tbilisi State Medical University, 33 Vazha-Pshavela Ave., 0177 Tbilisi, Georgia; 2 Curatio International Foundation, Tbilisi, Georgia; 3 Curatio International Consulting, Tbilisi, Georgia

## Abstract

**Background:**

Human resources (HR) are one of the most important components determining performance of public health system. The aim of this study was to assess adequacy of HR of local public health agencies to meet the needs emerging from health care reforms in Georgia.

**Methods:**

We used the Human Resources for Health Action Framework, which includes six components: HR management, policy, finance, education, partnerships and leadership. The study employed: (a) quantitative methods: from September to November 2004, 30 randomly selected district Centers of Public Health (CPH) were surveyed through face-to-face interviews with the CPH director and one public health worker randomly selected from all professional staff; and (b) qualitative methods: in November 2004, Focus Group Discussions (FGD) were held among 3 groups: a) 12 district public health professionals, b) 11 directors of district public health centers, and c) 10 policy makers at central level.

**Results:**

There was an unequal distribution of public health workers across selected institutions, with lack of professionals in remote rural district centers and overstaffing in urban centers. Survey respondents disagreed or were uncertain that public health workers possess adequate skills and knowledge necessary for delivery of public health programs. FGDs shed additional light on the survey findings that there is no clear vision and plans on HR development. Limited budget, poor planning, and ignorance from the local government were mentioned as main reasons for inadequate staffing. FGD participants were concerned with lack of good training institutions and training programs, lack of adequate legislation for HR issues, and lack of necessary resources for HR development from the government.

**Conclusion:**

After ten years of public health system reforms in Georgia, the public health workforce still has major problems such as irrational distribution and inadequate knowledge and skills. There is an urgent need for re-training and training programs and development of conducive policy environment with sufficient resources to address these problems and assure adequate functionality of public health programs.

## Background

The World Health Report 2006 documents the widespread health workforce crisis across the globe [1]. Similar to other regions and specialty areas, there has been a shortage of well trained public health workers in the Central and Eastern Europe/New Independent States (CEE/NIS) region as well [2].

Public health is defined as the science and art of preventing disease, prolonging life and promoting health, through the organized efforts of society. It has a population rather than an individual focus and involves mobilizing local, regional, national and international resources to ensure the conditions in which people can be healthy [3,4]. Performance of the public health system depends on multiple factors, among which human resources (HR) are one of the most important components [5]. The public health workforce requires up-to-date knowledge and skills to deliver essential public health services. To meet the training and continuing education needs of an evolving workforce, a clearer understanding of the functions and composition of the public health workforce both now and for the future is required [6].

Under the Soviet era, a highly centralized 'San-Epid' (sanepid) network focusing on environmental and epidemiological health was put in place in Georgia. Perhaps the most tangible achievement of the sanepid system was high immunization coverage and communicable disease control; however, it was relatively ineffective in combating environmental pollution, occupational diseases and noncommunicable diseases [2]. Public health workers employed by the sanepid system were mainly graduates from sanepid faculties of State medical institutes/universities, having completed a five-year course largely focusing on environmental health and infectious disease epidemiology and control. Graduation was followed by centralized training within continuous professional education programs. The 5-year course also included basic medical education (anatomy, physiology, biochemistry, etc.) as well as some clinical training (internal medicine, infectious diseases, surgery, etc.), and graduates were given an MD degree with specialization in epidemiology, environmental health, nutrition, etc. Sanepid system was a part of the entire health care system under the Ministry of Health umbrella management, and human resources were mainly managed by employing Soviet command and control approaches and style. Reporting lines were clearly defined under regulations of the Ministry of Health, and there was a strong accountability framework put in place.

Following independence, Georgia embarked on health sector reforms, the main focus of which was a modernization of public health services combined with decentralization efforts. In 1996, a dedicated Department of Public Health (DPH) was established to give greater emphasis to health promotion and disease prevention, ensure sufficient budget allocation for health promotion activities and encourage innovative community initiatives contributing to prevention of diseases [7]. The role of the DPH, which is part of the Ministry of Labour Health and Social Affairs (MoLHSA), was to monitor and assess the epidemiological situation of the population and to promote good health through education and management of preventive health services. In line with the decentralization policy implemented in the country, the MoLHSA supported establishment of local centers of public health (CPH), responsible for implementing public health activities on a district level [8].

Ten years later, after the initiation of health sector reforms in Georgia, investments in system building innovations had not resulted in sustainable health gain: over the past decade there has been a substantial increase in the incidence of sexually transmitted diseases, drug abuse, cardiovascular diseases, cancer, injuries as well as prevalence of smoking [9-11].

Recognizing the diversity of factors influencing unfavourable population health status, including low budget allocations to public health, weaknesses in organizational structure, poor legislation, lack of stewardship, the gap between private and public medicine, etc. [12,13], it could be argued that having an inadequate public health workforce (given that in Georgia, public health workers are mainly physicians, – i.e. graduates of sanepid faculties – 'public health workforce' here is defined as physicians providing essential public health services to promote physical and mental health and prevent diseases, injury, and disability), which has not been successful in assuming new roles and responsibilities, is also a significant contributing factor. The problem might be that while embarking on reforms there was no clear understanding of what are the competing needs for workforce supply in public health programs and activities considering the new mission, i.e. giving greater emphasis to health promotion and diseases prevention? What resources are available at central and district level? How does the new mission fit with human resource existing capacities? What do we need to do? This is an incomplete list of important questions that have to be answered to enable the public health system to deliver the necessary interventions.

The aim of this study was to assess adequacy (which denotes numbers, necessary skill mix, and adequate geographical distribution) of human resources of public health relative to the needs of the system under health care reforms. To the best of our knowledge, it is the first study in Georgia which has assessed the current status of the public health system by analysing the influence of multiple factors of human resources development, supply and distribution.

## Methods

### Study design

The study is comprised of two components: a) quantitative study – survey of centers of public health, and b) qualitative study – Focus Group Discussions (FGD) of selected professionals. From September to November 2004, 30 randomly selected, local CPH (of total of 66 in the country) were approached to collect quantitative data. Face-to-face interviews were conducted with CPH directors, who are most knowledgeable about staffing, educational background and professional experience of the employed public health workers and available resources and organizational structure of their units.

For the quantitative survey we used adapted questions from the Management Science for Health's HR Management Assessment Tool [14]. In each CPH, the survey collected data on public health workers demography and employment history as well as directors opinions (measured on a five point Likert scale) on HR management practices, available policies, funding levels and education attainment of the professional staff.

Alongside a director, in each CPH, one public health worker was randomly selected from all professional staff working at the facility and detailed information about training courses undertaken over the past three years in epidemiology, biostatistics, health policy and planning, health information systems, disease surveillance, etc., was obtained. The survey also enquired about additional credentials (e.g. certificates and diplomas) for self reported data verification.

For data analysis we used the recently elaborated Human Resources for Health Action Framework that includes six components – HR management, policy, finance, education, partnerships, and leadership, which is expected to enable countries in developing a concrete national health workforce strategy that could be supported and implemented in a planned and systematic manner [15].

Quantitative data were analysed by SPSS software. The main analysis was descriptive.

The Qualitative study was implemented in November 2004 through FGDs among local public health professionals and policy makers at central level. In total, three focus groups were formed: a) 12 district public health professionals (4 from rural districts, 4 from urban districts, and 4 from larger cities/regional centers), b) 11 directors of district public health centers (4 from rural districts, 4 from urban districts, and 3 from larger cities/regional centers), and c) 10 policy makers at central level (representatives of the Parliament of Georgia, MoLHSA units such as Department of Public Health, Department of Health Policy, and Department of Regulations).

Separate FGD guides were developed for each group. Questions aimed at understanding opinions regarding HR management (e.g. the vision and plans for HR development), policy (e.g. whether legislation and regulations support public health system to have well trained staff), finance (e.g. adequacy of resources to satisfy training needs), education (e.g. relevance of training courses to activities and skills required by public health programs). Furthermore, special inquiries were made concerning factors influencing the hiring and retention of trained professionals. Discussions also captured professional staff/directors' and policy maker's views about possibilities to address emerging challenges in the HR area. External facilitators managed the FGD and created a comfortable environment for participants to openly voice their opinions.

FGDs were audio taped and transcribed. The standard methodology was applied for qualitative analysis[16]. Notes were translated into English and selected quotations are presented in the text.

The study protocol was approved by the Institutional Review Board of Tbilisi State Medical University. Participants were included in the study only after obtaining written informed consent.

## Results

### Quantitative results

There were no refusals from the survey respondents (30 CPH directors and 30 randomly selected employees – one per CPH). The total number of public health workers employed at the surveyed 30 CPHs was 277, out of which 85% were females. The mean age of these public health workers was 43.3 years. The average number of public health workers per CPH was 9.2, ranging between 2 and 16 persons. The average length of working experience in their current public health job was 11.15 years, ranging between 3.6 and 26.5 years (Table 1). The distribution of professional staff varied across studied CPHs – for example, 10 remote rural district CPHs (33.3%) did not have any epidemiologists, whereas 5 urban district CPHs employed five and more epidemiologists.

**Table 1:** CPH public health workers demographic and employment data

CPH public health workers demographic and employment data	(N = 277)	95% CI	Min; Max
Mean age of public health workers	43.3	41.5–45.1	32; 53
Share of females among public health workers	236 (85%)		
Average number of public health workers per CPH	9.2	7.8–10.6	2; 16
Average length of working experience in current public health job (years)	11.1	9.1–13.2	3.6; 26.5

Assessment of HR policy, management, and funding issues at the CPH level showed that CPH directors, on average, neither disagreed nor agreed that in Georgia, the Public Health System has a coherent vision for HR development. They agreed that there are no budgetary provisions for HR development activities in their CPH. Overall, CPH directors agreed that there is no staff specifically charged with responsibility for HR functions, and no annual HR development plans exist in their CPH, whereas they disagreed that in their CPH they have an organizational policy manual, all members of staff have job descriptions, and formal staff evaluation is performed regularly (Table 2).

**Table 2:** CPH directors' judgment on various statements about HR policy, management, and funding issues in their CPH

Statements about HR policy, management, and funding issues in CPH	Mean values and SD from Likert scale
In Georgia, Public Health System has a coherent vision for HR development	3.27 (1.05)
Our CPH has a coherent vision for HR development	3.20 (1.06)
There is special budget allocated for HR development activities in our CPH	1.60 (0.56)
No staff specifically charged with responsibility for HR development functions in our CPH	4.00 (1.20)
No annual HR development plan exists in our CPH	4.33 (0.55)
Employee data (# of staff, skill/education level, etc) exists in our CPH	4.46 (0.51)
In our CPH personal folders exist for each staff member	4.50 (0.58)
Our CPH has its organizational policy manual	2.37 (0.76)
All members of staff in our CPH have job descriptions	2.50 (0.97)
Formal staff evaluation is performed regularly at our CPH	2.03 (0.81)

Note: (Likert scale values: 5 = strongly agree; 4 = agree; 3 = neither disagree nor agree; 2 = disagree; 1 = strongly disagree).

All 30 selected centers were implementing four federal programs financed by the state: a) infectious disease surveillance; b) health promotion; c) immunization program monitoring; and d) malaria prevention. When CPH directors were asked about the relevance of public health workers' knowledge and skills against the activities required by these programs (i.e. needs assessment, program planning, monitoring and evaluation, data analysis, fin/admin management), CPH directors, on average, neither disagreed nor agreed that public health professionals possessed sufficient skills and knowledge (Table 3).

**Table 3:** CPH directors' judgment on various statements about skills and knowledge necessary for the implementation of four federal programs

Our CPH public health professionals possess sufficient knowledge and skills for:	Mean values and SD from Likert scale			
	Health promotion	Disease surveillance	Immunization monitoring	Malaria prevention
a. Needs assessment	2.64 (0.64)	2.57 (0.63)	2.57 (0.63)	2.63 (0.65)
b. Program planning	2.80 (0.71)	2.70 (0.70)	2.70 (0.70)	2.75 (0.68)
c. Monitoring & Evaluation	2.80 (0.65)	2.80 (0.66)	2.80 (0.66)	2.79 (0.66)
d. Data analysis	2.64 (0.64)	2.73 (0.64)	2.73 (0.64)	2.67 (0.64)
e. Fin/admin management	2.84 (0.75)	2.66 (0.81)	2.66 (0.81)	2.75 (0.85)

Note: (Likert scale values: 5 = strongly agree; 4 = agree; 3 = neither disagree nor agree; 2 = disagree; 1 = strongly disagree).

Among 30 randomly selected public health professionals (one person from each centre), 13 (43.3%) respondents did not take any training course over the past three years in public health, epidemiology, biostatistics, health policy and planning, health information systems, disease surveillance, etc.

### Focus group discussions

#### Policy environment

It was said in all three groups that there is lack of adequate legislation addressing HR needs for the public health system. Although, work has recently started on public health law, which is expected to address the HR challenges.

"We do not know any legal documents setting requirements for training and professional development of public health professionals..." (CPH public health worker)

"There is no legislation regulating HR development issues for public health system.

Two new laws are pending – one is the law on public health and second the law on

continuous medical education. We hope that these two laws will really improve the

situation..." (Policy maker at central level)

#### HR management

All respondents thought that MoLHSA had no clear vision for HR development issues when reforms were initiated. Policy makers are of the opinion that they have developed a vision for HR development in the public health area, although as of yet there is no concrete plan.

"We do not remember any plan or activity addressing HR development at the beginning of the reform. Maybe now it does exist at central level, however we do not know anything about it here..." (CPH public health worker)

"In reality, we are just making first steps to shape our vision and develop realistic plans to address pending HR issues within the system..." (CPH director)

"Yes, we definitely have a vision on HR development for the public health system, however it is not reflected yet in concrete plans. Although we realize that there is a great need in developing such plans in the nearest future..." (Policy maker at central level)

#### Funding

FGD participants were consistent in admitting financial resource deficiency and lack of adequate motivations for public health system workforce. Diverse factors such as insufficient funding, lack of local government's interest in services offered by CPH, poor motivations created for CPH staff, etc, all affect negatively HR issues for public health system (Half of the CPH funding comes from the central budget (salary supplement and operational costs for implementation of federal programs) and half from the local municipal budget (mainly covers the CPH staff base salaries). Training courses that are occasionally provided to public health workers are usually funded through various international donor funded programs, and are mostly free of charge for participants).

"Resources are very limited, especially within the municipal budget, which does not allow us to do much for training and development of our staff..." (CPH director)

"Major reason is lack of adequate funding for public health services. Originally it was up to the central level to staff local CPH. When responsibilities were delegated to districts, local governments became reluctant to finance CPH staff adequately..." (CPH director)

"There is no motivation for a public health professional to work in a remote rural or mountainous area. Maximum salary is 120 GEL a month (approximately US$ 65), which is definitely not enough, and no additional living expenses are provided. It is clear that one can not find good professional who would agree to go there on that money..." (Policy maker at central level)

"Due to low salaries, public health professionals do not have motivation to perform well. At present, there are limited job opportunities in Georgia; otherwise many of them would leave their current jobs..." (Policy maker at central level)

#### Education

Low capacity of national training institutions, lack of adequate training programs and lack of coherent further training were mentioned by all FGD participants. CPH professionals were not happy with the quality of training courses, often being a formality and/or place for corruption. Concerns were also expressed with regard to the reliance on donor funded episodic training courses, and their sustainability.

"There are neither good training institutions nor good training programs, though we are required to pass exams. There are only episodic training courses provided by international organizations..." (CPH public health worker)

(Note: In Georgia, public health professionals are required to pass exams for certification

"There were some post graduate training courses (duration 4 months) we had to undertake but it was just a formality and place for corruption – i.e. participants could pay some money, not attend the training and still receive a certificate..." (CPH public health worker)

"There are definitely some efforts and activities, but the problem is that they are episodic. Lectures and seminars that are offered do not have regular basis, and thus it is not enough to achieve good results..." (Policy maker at central level)

#### Partnerships

Lack of coordination between national level institutions and donor funded programs were mentioned as factors not contributing to HR development for public health system.

"There is often duplication in training programs offered within the framework of different programs. In other words, they focus on the same topics, whereas other important topics are not covered at all..." (CPH director)

#### Leadership

Linkage of the CPH staff with the rest of the system is very poor. There are no organizational links with either public or private healthcare facilities, and there is no clear format for managing supervising health facilities and providers [17]. In line with these observations, CPH directors stated that it is difficult for them to provide direction and leadership to healthcare facilities and providers. CPH managers are frustrated – they never manage to get support from the local government and to mobilize resources for HR development.

"I have to explain to them who we are and why our service is important for the population" (CPH director)

"We are tired of requesting support from the local government, therefore we do not attempt to request any more" (CPH director)

#### Proposed recommendations

A number of recommendations emerged from FGDs in support of HR development that included both practical measures for immediate action and strategies for medium to long term. As for immediate action, major emphases were placed on development of effective policies to adequately distribute public health professionals across the country and retain them in remote rural areas as well as increase the funding for public health services to augment the salaries of local public health professionals. For the medium to long term, the highest priority was given to the elaboration of a national plan for HR development within the public health system, development of appropriate training programs, and establishment of a school of public health offering a master of public health (MPH) course.

"The government should develop effective policy to adequately distribute public health staff across the country, including remote rural areas..." (Policy maker at central level)

"Funding for public health services should be increased. In the first place, this should be reflected in the salaries of local public health professionals..." (CPH director)

"In the long run, we may think about establishing a of school of public health, which would provide an MPH course..." (Policy maker at central level)

## Discussion

This study describes a wide array of challenges that hinder HR development for public health system in Georgia. Our results are limited to the extent that respondents were frank and open. For example, the focus group context is important to consider because it affects the comfort level of participants and, consequently, how openly they respond to questions^16^. However, findings of FGD, e.g. lack of good training institutions and programs, are consistent with the results of the quantitative study showing that CPH professional staff do not possess sufficient skills and knowledge necessary for the implementation of public health programs.

Our results suggest that after ten years of health system reforms in Georgia, the current public health system has major deficiencies such as unequal distribution and low technical competence of public health workers, as well as poor HR management practices at district CPH. The reasons determining this inadequacy might include lack of adequate legislation, lack of vision and clear policies for HR development, limitations in funding, low technical capacity of national training institutions, lack of coordination between national level institutions and donor funded programs, lack of leadership, etc. It should be said that the challenges identified by this research have also been documented elsewhere in the countries of CEE/NIS. Namely these challenges include: inadequate funding, weak legislative framework, weak organization of services, etc. Taken together, these deficiencies have had a negative impact on the public health workforce, manifested in low qualification of public health workers [2]. Globally speaking, it is of interest that while Georgia has not suffered from the AIDS pandemic, the results seem to be the same as in other countries severely affected by AIDS – poor motivation and performance due to poor management and lack of investment in an efficient HR infrastructure [18].

Results of our study indicate that there may be an urgent need in improving the knowledge and skills of public health professionals in Georgia. Similar needs were successfully addressed in several countries in the region, where schools of public health have been established [2,19,20].

It should be said that research results helped to identify new directions for strengthening the public health system through HR development in Georgia.

### New law on public health

Firstly, the country embarked on development of a new public health law, which was passed by the Parliament in June 2007. The law has a special section on human resources, which defines MoLHSA roles and responsibilities in the development of human resources as well as requirements for education and continuous professional development of public health professionals.

### School of Public Health

The second important initiative has been the establishment of the school of public health under the auspices of the Tbilisi State Medical University. Involvement of the cadre of young Georgian professionals educated abroad and setting up an international partnership with leading international public health training centers have been the main strategic approaches that have been employed for establishing the Georgian School of Public Health.

However, without adequate planning for the number and type of staff to be produced by these institutions and without designing appropriate incentives for staff retention and motivation as well as improved HR management practices, the impact of new policies and improved education would be marginal. Workers alone are not panaceas. Building a high-performance workforce demands a hard, consistent, and sustained effort. For workers to be effective they must have necessary inputs, and for them to use these inputs efficiently they must be motivated, skilled, and supported [21]. Our study highlighted these needs, thus the Government of Georgia has to make further efforts to tackle the health workforce problems for public health.

## Conclusion

After ten years of health system reforms in Georgia, the current public health system has major deficiencies such as unequal distribution and low technical competence of public health workers, as well as poor HR management practices at district centers of public health.

There seems to be an urgent need for improving the knowledge and skills of public health professionals in Georgia. One of the solutions to successfully addressing these problems might be the establishment of the school of public health.

This should be accompanied with adequate planning for the number and type of staff to be produced by this institution and designing appropriate incentives for staff retention and motivation, as well as improved HR management practices at local centers of public health.

## Competing interests

The authors declare that they have no competing interests.

## Authors' contributions

MD and GG conceived the research and study design, lead the analysis and drafted the manuscript. GM and GM contributed to study design and assisted with reviewing the manuscript. All authors read and approved the final manuscript.
